# Sorting genomes with rearrangements and segmental duplications through trajectory graphs

**DOI:** 10.1186/1471-2105-14-S15-S9

**Published:** 2013-10-15

**Authors:** Mingfu Shao, Yu Lin, Bernard Moret

**Affiliations:** 1 Laboratory for Computational Biology and Bioinformatics, EPFL, Lausanne, Switzerland

## Abstract

We study the problem of sorting genomes under an evolutionary model that includes genomic rearrangements and segmental duplications. We propose an iterative algorithm to improve any initial evolutionary trajectory between two genomes in terms of parsimony. Our algorithm is based on a new graphical model, the trajectory graph, which models not only the final states of two genomes but also an existing evolutionary trajectory between them. We show that redundant rearrangements in the trajectory correspond to certain cycles in the trajectory graph, and prove that our algorithm converges to an optimal trajectory for any initial trajectory involving only rearrangements.

## Introduction

Genome-scale evolutionary events can be divided into two categories: genomic rearrangements and content-modifying operations. Genomic rearrangements shuffle gene orders and change gene orientations; they include inversions, transpositions, block exchanges, circularizations, and linearizations, all of which act on a single chromosome, and translocations, fusions, and fissions, which act on two chromosomes. All of these operations can be modelled in terms of the double-cut-and-join (DCJ) operation [1,2], which has formed the basis for much algorithmic research on rearrangements over the last few years [3-6]. Content-modifying operations, which affect both the number of gene copies and the gene orders, include insertions, deletions, and duplications.

A basic problem in phylogenetic inference is to compute an edit sequence between two genomes, i.e., a most parsimonious series of evolutionary operations that can transform one genome into the other. Many algorithms have been proposed for various edit problems under different evolutionary models and different assumptions about the genomes. Most of these algorithms use the same underlying data structure, the *breakpoint graph*, introduced by Bafna and Pevzner to study the edit problem under unsigned inversions [7,8]. In 1995, Hannenhalli and Pevzner gave the first polynomial-time algorithm to compute the edit distance (the length of the edit sequence) under signed inversions for unichromosomal genomes [9], which was later improved to linear time [10]. For multichromosomal genomes, the edit distance under the Hannenhalli-Pevzner model (signed inversions and translocations) has been studied through a series of papers [9,11-13], culminating in a fairly complex linear-time algorithm [3]. Under DCJ operations, Bergeron *et al. *[1] gave a simple linear-time algorithm to compute the edit distance, based on a slightly different representation of the rearrangements, using an *adjacency graph*. All of these algorithms consider only rearrangements and thus also assume equal gene content and no duplicate genes; consequently, their corresponding adjacency graphs have a very simple structure--a set of independent cycles and paths, making it possible to design efficient algorithms. Adjacency graphs have also been extended to study rearrangements with insertions and deletions [6,14,15], whole-genome duplications [16,17] as well as incorporating sequence information [18].

Segmental duplications have long been recognized as major driving forces of evolution [19,20]. In human genomes segmental duplications are hotspots for non-allelic homologous recombination leading to genomic disorders, copy-number polymorphisms, and gene and transcript innovations [21]. Kahn and Raphael gave an efficient dynamic programming algorithm to compute the duplication distance, in which rearrangement operations are not allowed [22], work later extended by introducing likelihood techniques and used to analyze the evolutionary relationships between duplication blocks [23]. Combining general segmental duplication with rearrangements, however, has remained an open problem, in spite of considerable work on combining rearrangements with content-modifying operations.

El-Mabrouk [24] extended the HP approach to include insertions and deletions, by providing an exact algorithm to compute edit distances for inversions and losses and also a heuristic to approximate edit distances for inversions, losses, and nonduplicating insertions; this approach was extended and refined by Marron *et al. *[25]. Yancopoulos *et al. *[6] proposed a model for calculating the edit distance under DCJ, single-gene insertions, single-gene deletions, and duplications. Lin *et al. *[26] proposed a new evolutionary model that integrates gene duplications and losses with genome rearrangement. Shao *et al. *[27] gave an approximation algorithm to compute the edit distance for two genomes with duplicated genes under a model that includes DCJ, insertions and deletions. A maximum likelihood approach has also been proposed to infer the evolutionary tree on whole-genome data under a model that considers both rearrangements and gene-content modifying events [28]. Most of this work, however, focused on distance computations based on the final states of the genomes and so cannot support identification of the actual sequence and location of any segmental duplications.

In this paper, we propose an iterative algorithm to refine any initial evolutionary trajectory between two genomes with rearrangements and segmental duplications. We introduce a new graphical data structure, the *trajectory graph*, to model any given evolutionary trajectory between two genomes. We begin by defining and illustrating the trajectory graph, then show correspondences between redundant rearrangements in the initial trajectory and certain cycles in the corresponding trajectory graph, and provide an effective algorithm to remove these redundant rearrangements by iteratively resolving the *active *(will be defined in the next section) cycles in the trajectory graph. We also prove that this algorithm converges to an optimal trajectory from any initial trajectory when the model is restricted to rearrangements.

## The trajectory graph

We use the notation introduced by Bergeron *et al. *[1]. Each genome is represented as a set of chromosomes, while each chromosome is a linear or circular list of genes. Each gene is represented by a nonzero integer, where the sign of the integer codes the orientation of that gene along the chromosome. The two ends of a gene *g *are called *extremities*, the head denoted *g_h _*and the tail *g_t_*. A gene *g *always points from *g_t _*to *g_h_*, i.e., we have *g *= *g_t _*→ *g_h _*and −*g *= *g_h _*→ *g_t_*. Two consecutive genes *a *and *b *can be connected by one *adjacency*, denoted as (*a*, *b*). Each adjacency can also be represented by the set of the two adjacent extremities. For example, adjacency (1, 2) can be written as {1*_h_*, 2*_t_*}, and adjacency (1, −2) can be written as {1*_h_*, 2*_h_*}. Note that (*a*, *b*) and (*b*, *a*) are two different adjacencies, while (*a*, *b*) and (−*b*, −*a*) are the same. Each linear chromosome ends with two single-extremity sets, to which we add a special *null extremity*, specified by 0, to form two normal adjacencies [6,27]. Thus, a linear chromosome with *n *genes has *n *+ 1 adjacencies, while a circular chromosome with *n *genes has *n *adjacencies. The *adjacency set *of a genome is defined as the set of all adjacencies in the genome. If all genes in the genome are distinct, the genome is uniquely represented by its adjacency set.

In our evolutionary model, we study two kinds of operations: DCJ and segmental duplication. A *DCJ *operation makes two cuts in the genome, producing four cut ends, and then rejoins them to produce two possible outcomes (a third "repairs" the cuts and thus makes no change). We can view a DCJ operation as a function of adjacencies: the input is two adjacencies (*a*, *b*) and (*c*, *d*) and the output is two new adjacencies (*a*, *d*) and (*c*, *b*), or (*a*, -*c*) and (-*b*, *d*). A *segmental duplication *operation duplicates a segment (a substring of the genome) and either creates a new circular chromosome out of the copy or inserts the copy in the genome at some location outside the original segment. A segment consisting of *n *genes *g*_1_, *g*_2_, ..., *g_n _*can be represented by its *n *- 1 adjacencies, (*g*_1_, *g*_2_), (*g*_2_, *g*_3_), ..., (*g*_*n-1*_, *g_n_*). To keep notation shorter, we will simply write (*g*_1_, *g*_2_, ..., *g_n_*) to represent these *n *- 1 adjacencies and, when appropriate, we simply use g' to represent the copy of the original gene *g *after a duplication.

We now define the two types of segmental duplications in term of adjacencies. The first type of the segmental duplication [19,20] inserts a copy of a segment (*g*_1_, *g*_2_, ..., *g_n_*) to a position specified by the adjacency (*a*, *b*); thus it takes (*g*_1_, *g*_2_, ..., *g_n_*) and (*a*, *b*) as input, and outputs $\left(a,\;g_{1}^{\prime }\right),\;\left(g_{1}^{\prime },\;g_{2}^{\prime },\cdots \;,\;g_{n}^{\prime }\right),\;\left(g_{n}^{\prime },\;b\right)$ and (*g*_1_, *g*_2_, ..., *g_n_*) or $\left(a,\;-g_{n}^{\prime }\right),\;\left(-g_{n}^{\prime },\;-g_{n-1}^{\prime },\cdots \;,\;g_{n-1}^{\prime }\right),\;\left(-g_{1}^{\prime },\;b\right)$ and (*g_1_*, *g*_2_, ..., *g_n_*). Note that $\left(g_{1}^{\prime },\;g_{2}^{\prime },\cdots \;,\;g_{n}^{\prime }\right)$ and $\left(-g_{n}^{\prime },\;-g_{n-1}^{\prime },\cdots \;,\;-g_{1}^{\prime }\right)$ represent the same adjacency set. The second type of the segmental duplication [29] creates a new circular chromosome with the copy of the segment (*g*_1_, *g*_2_, ..., *g_n_*), thus it takes only (*g*_1_, *g*_2_, ..., *g_n_*) as input and outputs $\left(g_{n}^{\prime },\;g_{1}^{\prime }\right),\;\left(g_{1}^{\prime },\;g_{2}^{\prime },\cdots \;,\;g_{n}^{\prime }\right)$ and (*g*_1_, *g*_2_, ..., *g_n_*).

**Figure 1:**
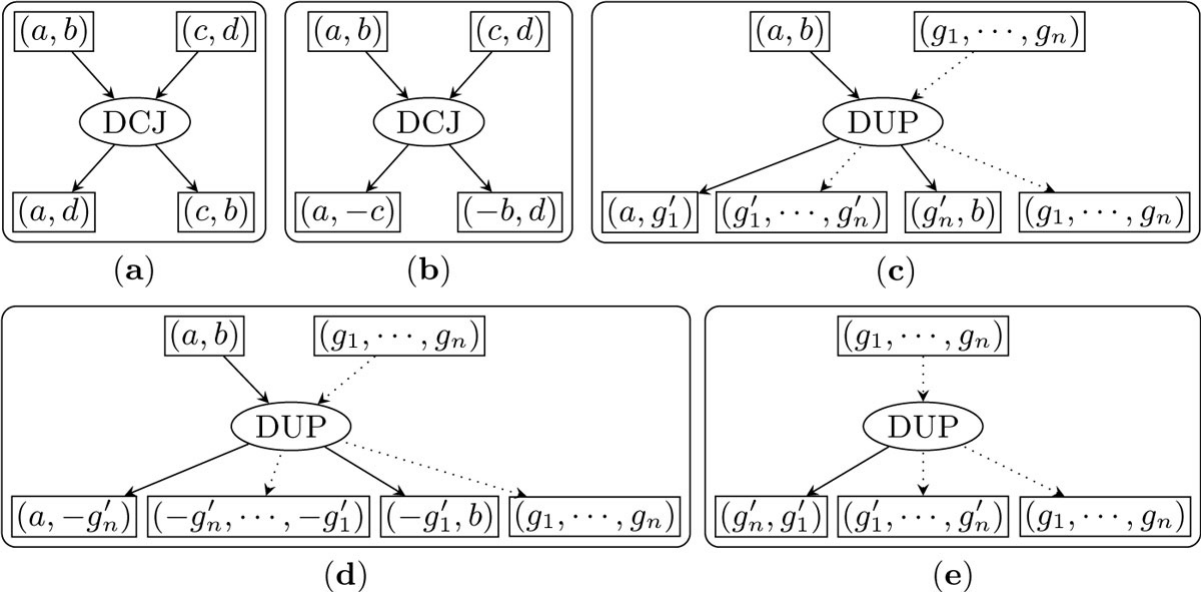
**Illustration of the DCJ and the segmental duplication as functions of adjacencies**. Part (*a*) and (*b*) are the DCJ, part (*c*) and (*d*) are the first type of the duplication, and part (*e*) is the second type of the duplication. Active edges are drawn with solid lines while inactive edges are drawn with dotted lines.

Each operation can be represented as a directed subgraph, composed of one *operation node *representing the operation itself, a number of *input adjacency nodes *(one for each of its input adjacencies), a number of *output adjacency nodes *(one for each of its output adjacencies), one directed edge from each input adjacency node to the operation node, and one directed edge from the operation node to each output adjacency node. Figure 1 illustrates these graph components for DCJ and the two types of duplication.

We say that an edge is *active *if the adjacency associated with the node to which it is attached has been changed by the operation, *inactive *otherwise. In the graph for a DCJ operation, all four edges are active. In the graph for the first type of duplication, only the edge from (*a*, *b*) to the operation node and the two edges from the operation node to the output adjacencies $\left(a,\;g_{1}^{\prime }\right)$ and $\left(g_{n}^{\prime },\;b\right)$, or $\left(a,\;-g_{n}^{\prime }\right)$ and $\left(-g_{1}^{\prime }\;,\;b\right)$ are active, while the other edges are inactive. In the graph for the second type of duplication, only the edge from the operation node to $\left(g_{n}^{\prime },\;g_{1}^{\prime }\right)$ is active; all other edges are inactive. We say that a cycle in the trajectory graph is an *active cycle *if all of its edges are active and define the *size *of a cycle as the number of the operation nodes it contains.

**Figure 2:**
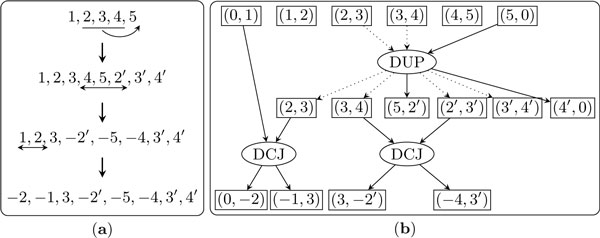
**A trajectory graph**. The initial genome consists of one linear chromosome of 5 genes, (1, 2, 3, 4, 5). The duplication operation inserts a copy of (2, 3, 4) to the right end, which transforms the genome into (1, 2, 3, 4, 5, 2', 3', 4'). Then two DCJ operations, one inverting the segment of (4, 5, 2') and the other inverting the segment of (1, 2), generate the final genome as (-2, -1, 3, -2', -5, -4, 3', 4'). Adjacency nodes (1, 2) and (4, 5) form two trivial connected components, while the rest of the graph forms a nontrivial connected component. Figure legend text.

Given two adjacency sets *X *and *Y*, a *sorting path P *= {*p*_1_, *p*_2_, ..., *p_n_*} from *X *to *Y *is a series of operations that transform *X *into *Y*. The *trajectory graph G*(*P*) with respect to *P *naturally delineates the input and output of each operation and the dependency relationships between them. Let *S_k _*be the adjacency set after sequentially performing operations *p*_1_, *p*_2_, ..., *p_k _*starting from *X*. The *trace *from *X *to *Y *with respect to *P *is (*X *= *S*_0_, *S*_1_, *S*_2_, ..., *S_n _*= *Y*). To construct *G*(*P*), the initial step is to draw one *adjacency node *for each adjacency in *X*. Then we sequentially handle each operation in *P*, ensuring that, before tackling operation *p_k_*, all adjacency nodes of outdegree 0 in the current graph are exactly *S_k-1_*. To add operation *p_k_*, we connect the component graph for operation *p_k _*to the current graph by replacing all the input adjacency nodes with their counterparts in the current graph. After all operations are added, the set of all adjacency nodes of outdegree 0 is then exactly *Y*. Figure 2 illustrates the construction.

By construction, the trajectory graph has the following two properties. First, the adjacency nodes of indegree 0 form the adjacency set of *X *and the adjacency nodes of outdegree 0 form the adjacency set of *Y*, while all other adjacency nodes have indegree 1 and outdegree 1. Second, the trajectory graph is a directed acyclic graph and any topological sorting of all the operation nodes is a valid sorting path from *X *to *Y*.

## An iterative algorithm to improve any trajectory

Given any two genomes and initial evolutionary trajectory *P *between them, we build the trajectory graph *G*(*P*) and give the following sufficient condition in *G*(*P*) to identify and resolve redundant rearrangements in *P*.

**Theorem 1**. *Let P be a sorting path from X to Y. If G*(*P*) *contains active cycles, then we can find another sorting path P' from X to Y with fewer DCJ operations and an equal number of duplications*.

*Proof*. Let *C *be an active cycle in *G*(*P*). Since *G*(*P*) is directed, we can represent *C *as two node-disjoint directed paths starting from the same *top node *and ending at the same *bottom node*. Clearly, neither of these two nodes can be an adjacency node: the top node must have outdegree 2 and the bottom node must have indegree 2. Moreover, the bottom node must be a DCJ node, since there is at most one active edge pointing to each duplication node. We now show how to exchange the bottom operation node with one of the two parent operation nodes, thereby moving one operation node out of *C*, until there are only two operation nodes left in *C*, which we can always replace with at most one operation.

We choose one of the two parent operation nodes to guarantee that the exchange will not create any directed cycles. If both parent nodes are independent (there is no directed path from one to the other), then we arbitrarily choose one; otherwise, we always choose the one that is on the directed path from the other to the bottom node.

**Figure 3:**
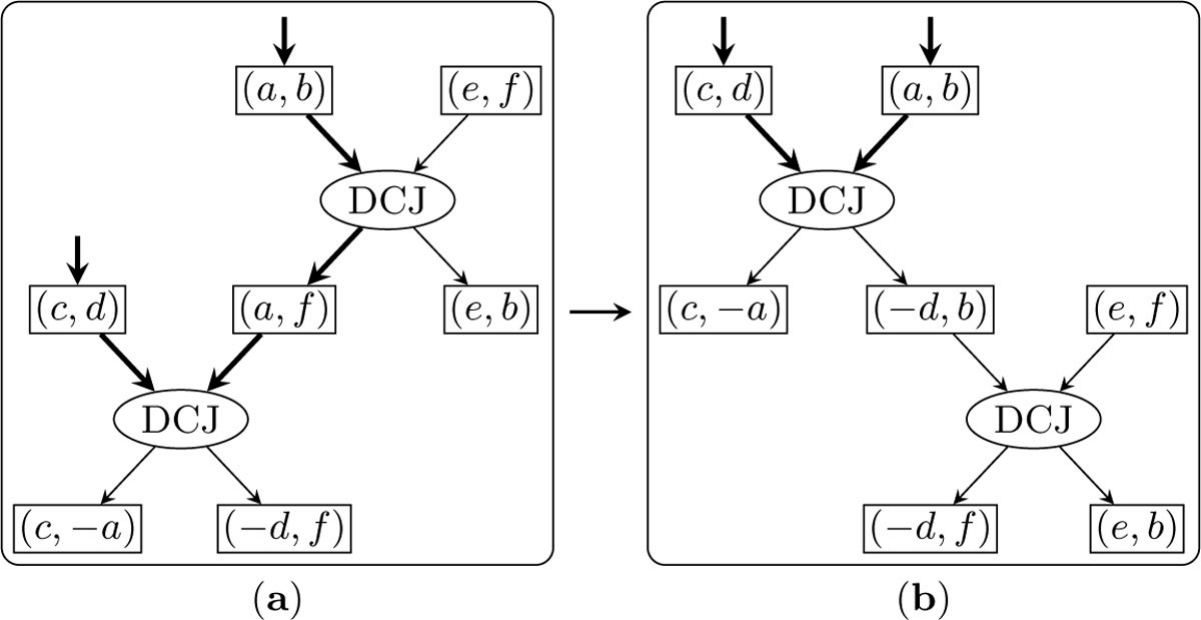
**Exchanging two DCJ nodes to reduce the size of the active cycle**. Edges in the active cycle are in bold.

Figure 3 shows how to exchange the bottom DCJ node with a parent DCJ node. We view the two DCJ nodes as a single supernode, with three input adjacencies and three output adjacencies. We replace the current two DCJ nodes with two new ones, keeping the inputs and outputs of the supernode unchanged. The new top DCJ node takes the two input adjacencies of the supernode that are linked in *C *as its inputs and outputs two adjacencies, one of which is among the outputs of the supernode. The new bottom DCJ node takes the other output adjacency of the new top DCJ node and the remaining input adjacency of the supernode as inputs and outputs the other two output adjacencies of the supernode. After the exchange, the new bottom DCJ node is out of the new active cycle, while the new top DCJ node becomes the bottom node of the new active cycle.

**Figure 4:**
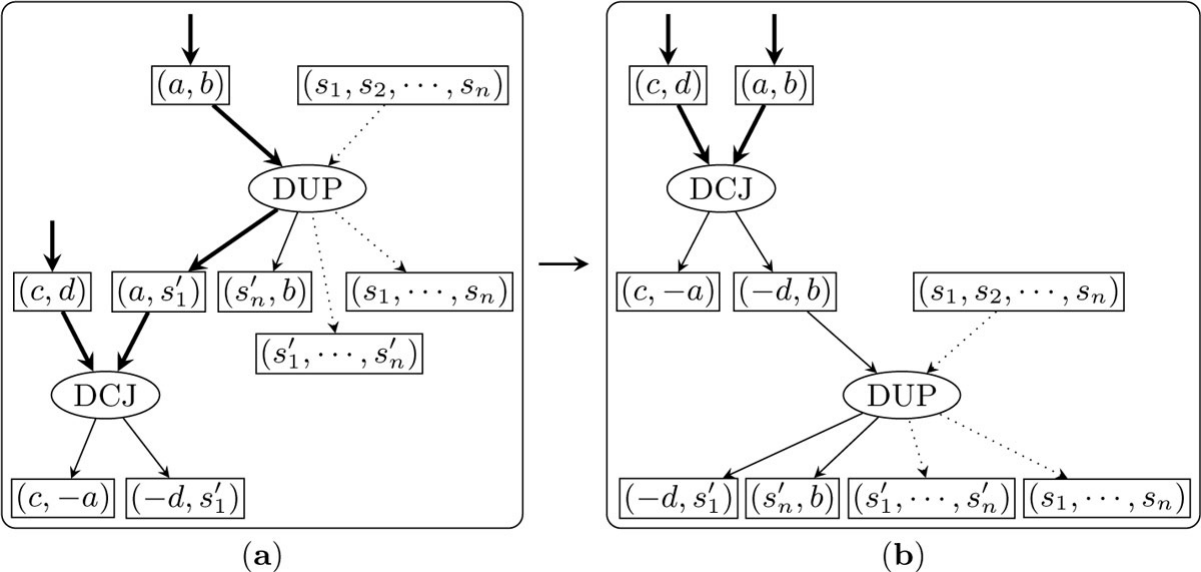
**Exchanging the bottom DCJ node with its parent duplication node**.

Figure 4 shows how to exchange the bottom DCJ node with its parent duplication node. Again, we consider these two operation nodes as a single supernode. The new DCJ node takes the two adjacency nodes linked in *C *as inputs and outputs two adjacencies, one of which is among the outputs of the supernode while the other is the insert position of the new bottom duplication node. After the exchange, the new bottom duplication node will be out of the new active cycle and the new parent DCJ node will be the bottom node of the new active cycle.

Through these exchanges, *C *will be reduced to just two operation nodes, the top one and the bottom one. Now we show that we can always replace these two operations with at most one operation.

**Figure 5:**
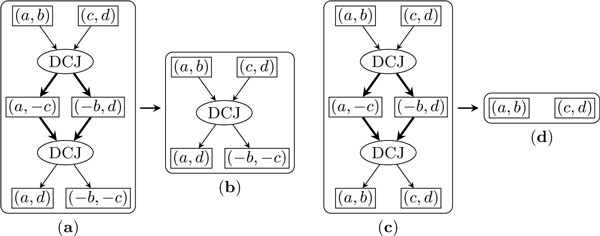
**Resolve the active cycle consisting of two DCJ operations**.

Consider first the case in which the two operation nodes are both DCJ operations. If the input and output of the supernode are different, then we can use one new DCJ node to connect them, as shown in Figure 5(*a*, *b*); otherwise, we do not need any operation node, as shown in Figure 5(*c*, *d*).

**Figure 6:**
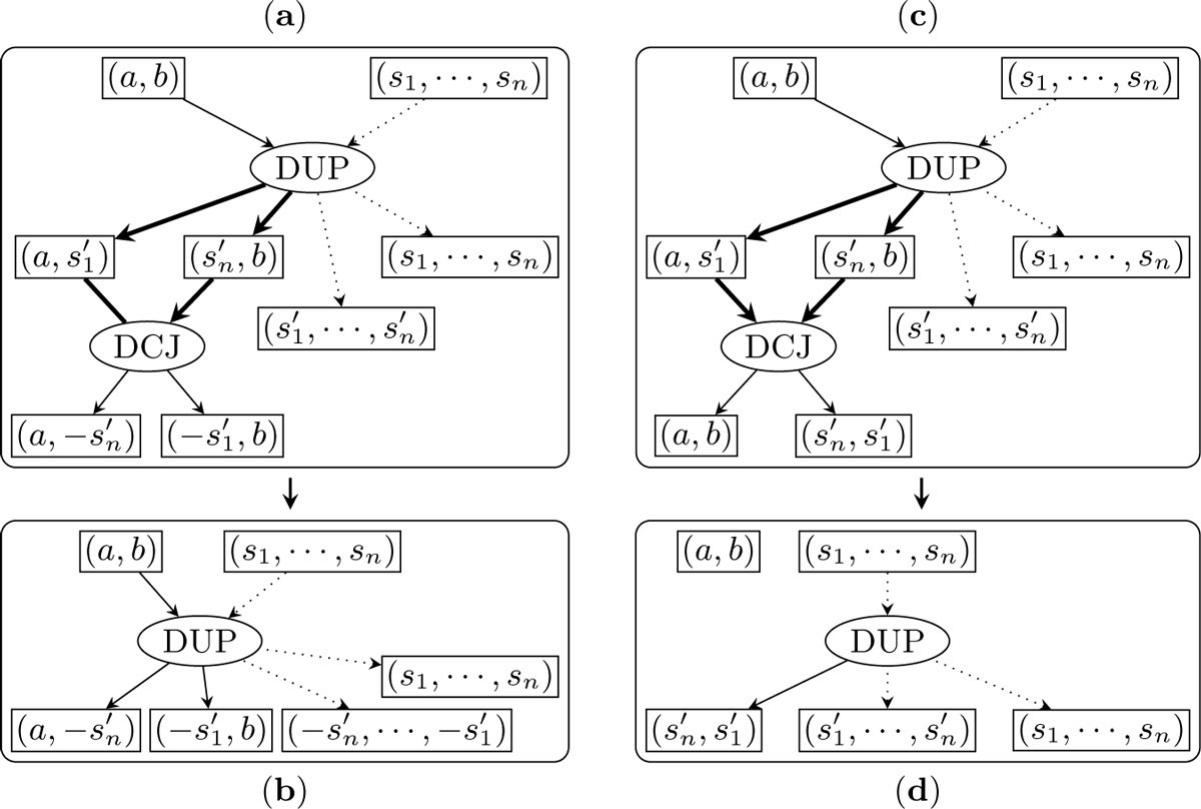
**Resolve the active cycle consisting of one DCJ operation and one duplication**.

Next consider the case in which the top node is a duplication and the bottom node is a DCJ. Assume the top duplication inserts a copy of the segment to position (*a*, *b*). If (*a*, *b*) is not one of the output adjacencies of the bottom DCJ node, then these two operations can be replaced by one duplication which inserts the inverted segment to the same position, as shown in Figure 6(*a*, *b*); otherwise, these two operations can be replaced by one duplication which creates a circular chromosome from the copy of the segment, as shown in Figure 6(*c*, *d*).

Note that the trajectory graph remains a directed acyclic graph. Thus we can retrieve one sorting path from any topological sorting of the operation nodes in the final trajectory graph; since we also showed that the number of DCJ operation nodes has reduced by at least one and the number of duplication nodes is unchanged, the theorem is proved.    □

Given the trajectory graph *G*(*P*) constructed from two input genomes and an initial trajectory *P*, our algorithm iteratively applies Theorem 1 to reduce the number of operations until there is no active cycle in *G*(*P*). This algorithm will always terminate since resolving each cycle will reduce the number of operation nodes in *G*(*P*) and such number is always non-negative.

## The trajectory graph restricted to rearrangements

We study the trajectory graphs with only DCJ operations. We show that the above iterative algorithm always converges to an optimal trajectory for any initial trajectory.

We first investigate the structure of the trajectory graphs under a rearrangement-only model and illustrate the close relationship with adjacency graphs. Recall that given two adjacency sets *X *and *Y*, the adjacency graph is defined as a bipartite multigraph *A *= {*X*, *Y*, *E*}, in which *u *∈ *X *and *v *∈ *Y *are linked by one edge if *u *and *v *share one extremity and by two edges if they share two extremities. If *X *and *Y *have the same extremity set and each extremity appears only once, the adjacency graph consists of node-disjoint cycles and the minimum number of DCJ operations needed to transform *X *into *Y *is |*X*| - *c*, where *c *is the number of cycles in *A *[1].

Let a trajectory *P *consist of only DCJ operations. For a connected component *C *of the trajectory graph *G*(*P*), we use *I*(*C*) to denote the set of adjacency nodes of indegree 0 in *C*, *O*(*C*) to denote the set of adjacency nodes of outdegree 0 in *C*, *A*(*C*) to denote the set of adjacency nodes with indegree 1 and outdegree 1, and *D*(*C*) to denote the set of DCJ nodes in *C*. We say that a connected component is *trivial *if it is a single adjacency node and *nontrivial *otherwise (for examples see Figure 2).

**Lemma 1**. *Let C be a connected component in the trajectory graph G*(*P*) *where P consists of only DCJ operations. Then we have C is a tree if and only if *|*D*(*C*)| = |*I*(*C*)| - 1.

*Proof*. If *C *is trivial, it is a tree, and we have |*D*(*C*)| = 0 and |*I*(*C*)| = 1, hence the lemma holds. Assume then that *C *is nontrivial. The number of edges equals the sum of indegrees, which is |*O*(*C*)|+ |*A*(*C*)|+ 2 · |*D*(*C*)|; the number of nodes is |*I*(*C*)| + |*O*(*C*)| + |*A*(*C*)| + |*D*(*C*)|, since *I*(*C*) and *O*(*C*) are disjoint when *C *is nontrivial. *C *is connected, so it is a tree exactly when it has one more vertices than it has edges, hence we can write |*O*(*C*)|+|*A*(*C*)|+2·|*D*(*C*)| = |*I*(*C*)|+|*O*(*C*)|+|*A*(*C*)|+|*D*(*C*)|-1, which yields |*D*(*C*)| = |*I*(*C*)|-1, as desired.    □

The following lemma shows that there is one-to-one correspondence between trees in the trajectory graph and cycles in the adjacency graph.

**Lemma 2**. *Let C be a tree in the trajectory graph G*(*P*) *where P consists of only DCJ operations. The corresponding adjacency graph A *= {*I*(*C*), *O*(*C*), *E*} *consists of exactly one cycle*.

*Proof*. If *C *is trivial, then *A *is a cycle of length 2, hence the lemma holds. For a nontrivial *C*, we proceed by contradiction. Since *C *is nontrivial, we have that *I*(*C*) and *O*(*C*) are disjoint. Besides, *I*(*C*) and *O*(*C*) have the same extremity set. Thus, *A *= {*I*(*C*), *O*(*C*), *E*} consists of node-disjoint cycles. Now suppose that *A *consists of two or more cycles. We partition *A *into two parts by taking one arbitrarily chosen cycle as the first part and the remaining cycle(s) as the second part. We then label all extremities in the first part as e_1 _and all extremities in the second part as *e*_2_. We divide the adjacency nodes in *C *into three categories: if its two extremities are both labeled *e*_1_, then label the node a_1_; if its two extremities are both labeled *e*_2_, then label the node *a*_2_; otherwise, label the node *a*_3_. Now we can classify any DCJ operation into one of the following 7 types:

1. {*a*_1_, *a*_1_} → {*a*_1_, *a*_1_}, or

2. {*a*_2_, *a*_2_} → {*a*_2_, *a*_2_}, or

3. {*a*_1_, *a*_2_} → {*a*_3_, *a*_3_}, or

4. {*a*_1_, *a*_3_} → {*a*_1_, *a*_3_}, or

5. {*a*_2_, *a*_3_} → {*a*_2_, *a*_3_}, or

6. {*a*_3_, *a*_3_} → {*a*_3_, *a*_3_}, or

7. {*a*_3_, *a*_3_} → {*a*_1_, *a*_2_}.

**Figure 7:**
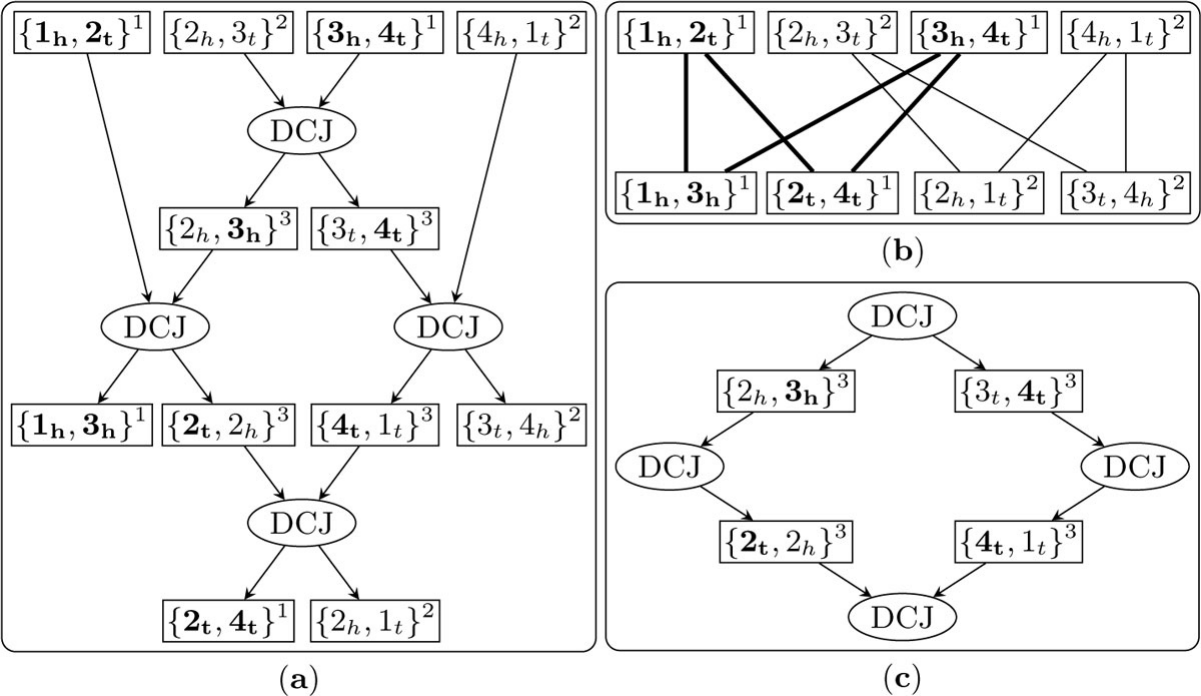
**An example for the proof of Theorem 2**. Part (*a*) shows a non-trivial connected component *C *of a trajectory graph. Part (*b*) is the corresponding adjacency graph *A *= {*I*(*C*), *O*(*C*), *E*}, which has two cycles. All extremities in the first cycle are shown bold. The superscripts 1, 2 and 3 on each adjacency represent labels of *a*_1_, *a*_2 _and *a*_3 _respectively. After removing all adjacency nodes in (*a*) labeled as *a*_1 _or *a*_2_, the remaining part is shown in part (*c*), in which all nodes have even total degree.

Note that the labels on the adjacency nodes in *I*(*C*) ∪ *O*(*C*) must be either *a*_1 _or *a*_2_, since these adjacencies are the nodes of the adjacency graph, whose two extremities have the same type. Note also that there must exist at least one adjacency node in *C *that is labeled as *a*_3_: otherwise the DCJ operation can be only of type (1) or (2) and *C *can be divided into two disconnected subgraphs, one defined by adjacency nodes labeled *a*_1 _and DCJ nodes of type (1), which contradicts the fact that *C *is connected. Now, remove all adjacency nodes labeled *a*_1 _or *a*_2 _and their adjacent edges: the remaining nodes must have even total degree--see Figure 7. The reason is that all adjacency nodes in *I*(*C*) ∪ *O*(*C*) are removed, while adjacency nodes in *A*(*C*) either are removed or are labeled as *a*_3 _and have degree 2. Moreover, all operation nodes must have even total degree, as easily verified by checking the 7 types. Hence there must be one cycle in *C*, a contradiction since *C *is a tree.    □

**Theorem 2**. *A trajectory P consisting of only DCJ operations is optimal if and only if the corresponding trajectory graph G*(*P*) *consists of trees*.

*Proof*. If *G*(*P*) contains at least one cycle *C*, then this cycle *C *must be an active cycle since all edges in *G*(*P*) are active for the DCJ-only model. Thus, according to Theorem 1, we have that *P *is not optimal.

We now prove that, if *G*(*P*) consists of only trees, then *P *is optimal. Now assume that *G*(*P*) consists of *m *trees, *T*_1_, *T*_2_, ..., *T_M_*. Applying Lemma 1 to each tree, we find that the total number of DCJ nodes in *G*(*P*) is

$$\overset{m}{\underset{k=1}{\sum }}|D\left(T_{k}\right)|=\overset{m}{\underset{k=1}{\sum }}|I\left(T_{k}\right)|-1=\overset{m}{\underset{k=1}{\sum }}|I\left(T_{k}\right)|-m.$$

On the other hand, according to Lemma 2, there are exactly *m *cycles in the adjacency graph $A=\left\{\cup_{k=1}^{m}I\left(T_{k}\right),\cup_{k=1}^{m}O\left(T_{k}\right),E\right\},$ which implies that the minimum number of DCJ operations needed is $\sum_{k=1}^{m}|I\left(T_{k}\right)|-\;m$. Thus *P *is optimal, as desired.    □

**Corollary 1**. *The iterative algorithm converges to an optimal trajectory from any initial trajectory*.

*Proof*. The iterative algorithm terminates when there is no active cycles in the trajectory graph. According to Theorem 2, any trajectory retrieved from the trajectory graph is optimal.    □

## Discussion and conclusion

Theorem 1 gives us a means to reduce the cost of a given sorting path. Unfortunately, the converse of the theorem does not hold: it is not hard to see how to take advantage of duplication nodes to produce a counterexample. Thus repeated applications of the constructive proof of Theorem 1 do not ensure optimality. However, the iterative improvement procedure can form the basis for strong heuristics or good approximation algorithms.

The trajectory graph naturally combines rearrangements and segmental duplications (or, in general, content-modifying operations) in a single model. Such a basis is crucial to the development of strong characterizations and good algorithms. We also took a step in that direction by showing that the trajectory graph is a proper augmentation of the adjacency graph, in terms of that both graphs can be used to obtain an optimal sorting scenario when only DCJ operations are considered, and by describing an efficient iterative algorithm that can be used on a more general model. Our current work focuses on using this improvement method within a large optimization framework (e.g. incorporating methods to find good initial trajectories) to derive fast and accurate approximations.

Different trajectories may correspond to the same trajectory graph, if they can be retrieved by different ways of topological sorting in the same graph. Thus the trajectory graph is useful in representing equivalent trajectories as well as characterizing the space of all optimal trajectories under both rearrangements and content-modifying operations (like the cases under inversions [30,31] and DCJ operations [32] on adjacency graphs), and thus forming a basis for future statistical analysis.

Under the DCJ model, if the two cuts are in the same linear chromosome, one of the two nontrivial outcomes is to circularize a segment of DNA as a circular chromosome (also called circular intermediate), which has recently been inferred in the evolution of cow genomes [33]. Recent evidence also showed that segmental duplications may also be mediated by circular intermediates in fish genomes [29]. The proof of Theorem 1 makes natural use of the connection between rearrangements and segmental duplications through circular intermediates, and thus may be useful to identify possible circular intermediates in the evolutionary trajectory.

## Competing interests

The authors declare that they have no competing interests.
